# Multivariate linear mixture models for the prediction of febrile seizure risk and recurrence: a prospective case–control study

**DOI:** 10.1038/s41598-023-43599-5

**Published:** 2023-10-13

**Authors:** Jan Papež, René Labounek, Petr Jabandžiev, Katarína Česká, Kateřina Slabá, Hana Ošlejšková, Štefania Aulická, Igor Nestrašil

**Affiliations:** 1 Department of Pediatrics, Faculty of Medicine and University Hospital, Masaryk University, Brno, Czech Republic; 2 Department of Pediatric Neurology, Faculty of Medicine and University Hospital, Masaryk University, Černopolní 9, Brno, 612 00 Czech Republic; 3 Division of Clinical Behavioral Neuroscience, Department of Pediatrics, University of Minnesota, Masonic Institute for the Developing Brain, 2025 East River Parkway, Minneapolis, MN 55414 USA; 4 Ondrej Slaby Research Group, Central European Institute of Technology, Masaryk University, Brno, Czech Republic

**Keywords:** Neurology, Computational models, Data processing, Predictive markers

## Abstract

Our goal was to identify highly accurate empirical models for the prediction of the risk of febrile seizure (FS) and FS recurrence. In a prospective, three-arm, case–control study, we enrolled 162 children (age 25.8 ± 17.1 months old, 71 females). Participants formed one case group (patients with FS) and two control groups (febrile patients without seizures and healthy controls). The impact of blood iron status, peak body temperature, and participants’ demographics on FS risk and recurrence was investigated with univariate and multivariate statistics. Serum iron concentration, iron saturation, and unsaturated iron-binding capacity differed between the three investigated groups (p_FWE_ < 0.05). These serum analytes were key variables in the design of novel multivariate linear mixture models. The models classified FS risk with higher accuracy than univariate approaches. The designed bi-linear classifier achieved a sensitivity/specificity of 82%/89% and was closest to the gold-standard classifier. A multivariate model assessing FS recurrence provided a difference (p_FWE_ < 0.05) with a separating sensitivity/specificity of 72%/69%. Iron deficiency, height percentile, and age were significant FS risk factors. In addition, height percentile and hemoglobin concentration were linked to FS recurrence. Novel multivariate models utilizing blood iron status and demographic variables predicted FS risk and recurrence among infants and young children with fever.

## Introduction

Febrile seizures (FS) are the most common convulsive disorder in childhood, usually associated with a fever of 38 °C (100.4°F) or higher and an incidence of 2–11%^1–4^. Fever is not triggered by metabolic disorders or central nervous system (CNS) infection; both etiologies need to be excluded in the differential diagnostic workup. Children aged four months to 5 years are mostly affected with the peak incidence at 18 months of age^3,4^. Besides an increased risk of epilepsy^5^ and psychiatric disorder^6^, the recurrent FS (RFS) represent the most common long-term effect of FS^4,6^ with an estimated 14–24% of the recurrence within the same febrile illness^2^. Overall, the recurrence decreases with age from 50% in children younger than 12 months at the first FS to 30% afterward. After a second FS, the probability of additional FS is 50%^3,4^. The accurate prediction of FS/RFS can outline strategies to prevent FS and mitigate the burden of FS on a child’s health or avoid repeated hospital visits, which may deepen the anxiety in children and families^1–4^.

Multiple FS/RFS risk predictors have been proposed such as body peak temperature, iron status, electrolyte imbalance, age, sex, and genetics, but inconsistent or contradictory results across various studies were observed. Combining more than one predictor may drive the risk prediction higher^2,7,8^, thus, estimating the FS/RFS risk with higher accuracy. Iron deficiency (ID) is frequent in infants and toddlers with the concurrent peak age as FS^7,8^. The association of iron status and febrile seizures (FS) has been postulated but supported by equivocal or inconclusive reports^1,7,9–11^. This controversy may be explained by cultural and geographic differences, as iron status is closely linked to socioeconomic status, malnutrition, and weaning practices^1,8–11^.

In this work, we collected blood iron status and demographic data in prospective cohorts of children with FS, RFS, fever without seizures, and afebrile healthy controls. Next, we investigated separation ability, i.e., sensitivity (SE) and specificity (SP)^12^, of individual variables. Then, we designed multivariate linear mixture models sensitive and specific to FS risk and recurrence.

## Methods

This prospective case–control study was performed at the University Hospital Brno, Czechia, from April 1, 2015 to August 31, 2017 with a subsequent 5-year follow-up (until August 31, 2022) under the Masaryk University Ethics Review Board approval and was conducted in accordance with the ethical principles of the Declaration of Helsinki. The informed consent form was obtained from every participant's parent/legal guardian prior to the study enrollment.

### Participants

A total of 162 Caucasian children were enrolled and formed one case group (FS group) and two control groups (febrile patients without seizures and healthy controls). Inclusion criteria were age 4–72 months, electroencephalograph (EEG) without epileptiform abnormality and normal background activity corresponding to age (FS group), normal neurodevelopment, and neurological exam. The diagnostic criteria of FS followed the American Academy of Pediatrics clinical guidelines^3,4^. The FS group consisted of 53 children (15 females) aged 4–70 months and formed two subgroups; non-recurrent FS (36 children, 11 females) and RFS (17 children, 4 females). Three children (one female) presented complex non-recurrent FS (one with repeated seizure within 24 h and two with transient focal post-ictal deficit); all the other FS children presented with a simple FS. Fifty-three children (26 females), aged 6–70 months, had a febrile illness caused by respiratory or urinary tract infection but without seizures. The healthy control group, recruited from children coming for a regular preventive care exam, comprised 56 children (30 females) aged 6–67 months. Exclusion criteria were age below four or above 72 months, peak body temperature ≤ 37.5 °C (99.5°F) for febrile groups, psychomotor developmental delay, malnourishment, seizures lasting more than 15 min, focal signs or lateralization in a neurological exam, epilepsy, genetic epilepsy with febrile seizures plus, antiepileptic-drug usage, history of afebrile seizures, history of CNS infection or severe head trauma, electrolyte, glucose, or homeostasis imbalance. Children suffering from chronic illnesses such as cardiovascular, renal, rheumatological, or malignant diseases, hemoglobinopathies, or other blood disorders that are associated with a higher likelihood of anemia were excluded. Demographics are summarized in Table 1.

**Table 1:** Demographic and blood iron status variables.

Demographics	FS			Non-recurrent FS			Recurrent FS			Febrile controls			Healthy controls		
Subjects (n, %)	53			36	67.9%		17	32.1%		53			56		
Females (n, %)	15	28.3%		11	30.6%		4	23.5%		26	49.1%		30	53.6%	

	Median	Mean	SD	Median	Mean	SD	Median	Mean	SD	Median	Mean	SD	Median	Mean	SD
Descriptive metric															
Age (months)	19	23.9	14.6	17	21.5	14.9	24	26.5	14.0	24	30.2	19.9	33	30.5	16.7
GA (weeks)	40	39	1.4	40	39.1	1.5	40	38.9	1.3	39	39.3	1.6	40	39.8	1.1
Height (percentile)	54.5	52.1	30	62.5	62	26.3	31.5	41.6	30.4	40	43.2	24.6	50	48.6	22
Weight (percentile)	37.5	42.4	31.5	51.0	50.8	31.9	22.0	33.6	29.0	40.0	43.2	24.4	50.5	49.3	22.9
Age at the first seizure (months)	16	20.1	13.6	17	21.5	14.9	15	17.1	9.9	n/a	n/a	n/a	n/a	n/a	n/a
Peak temperature (°C)	38.6	38.7	0.6	38.6	38.8	0.6	38.6	38.6	0.6	38.4	38.5	0.6	n/a	n/a	n/a
Iron status															
RBC (10e6/μL)	4.57	4.61	0.58	4.53	4.50	0.37	4.62	4.83	0.83	4.59	4.53	0.37	4.61	4.61	0.31
HGB (g/L)	115.50	116.20	10.00	114.00	113.80	10.80	117.50	118.70	8.40	120.00	119.30	10.30	121.50	121.30	8.30
Fe (μmol/L)	3.50	3.99	1.76	3.00	3.81	1.90	3.80	4.34	1.42	8.60	9.07	5.68	13.70	13.84	7.19
Fer (ng/mL)	41.90	52.15	43.86	43.10	57.81	49.97	36.90	40.84	25.55	38.60	57.74	47.59	25.45	36.09	36.94
TF (g/L)	2.68	2.75	0.42	2.73	2.76	0.47	2.63	2.73	0.30	2.54	2.56	0.38	2.82	2.83	0.47
satFe (%)	5.00	5.78	2.53	5.00	5.44	2.62	6.00	6.44	2.25	13.00	14.17	8.32	20.00	19.77	9.67
UIBC (μmol/L)	63.79	66.07	11.88	65.34	66.92	12.54	60.99	64.38	10.58	53.80	54.06	10.39	56.59	56.46	13.10

*GA* gestational age, *RBC* red blood cells, *HGB* hemoglobin, *Fe* serum iron concentration, *Fer* serum ferritin concentration, *TF* serum transferrin concentration, *satFe* iron saturation, *UIBC* unsaturated iron binding capacity, *FS* febrile seizures, *n/a* not applicable.

### Data collection

Each participant underwent a blood draw with the analysis of red blood cell count (RBC), hemoglobin (HGB), serum iron (Fe), iron saturation (satFe), ferritin (Fer), transferrin (TF), and unsaturated iron-binding capacity (UIBC). In FS and RFS patients, electrolytes and vitamin D were also measured. Blood draw analysis results, peak body temperature, age, sex, gestational age (GA), height and weight percentiles were utilized in between-group difference testing and multivariate statistical modeling. In addition, all available screening values for all seizures were reported in participants with RFS. The precise FS duration and interval between fever onset and FS were not collected as these parent-reported outcomes tend to be inaccurate. The study participants were followed up for five years to record RFS or treatment for ID or anemia.

### Statistical analysis

**Figure 1:**
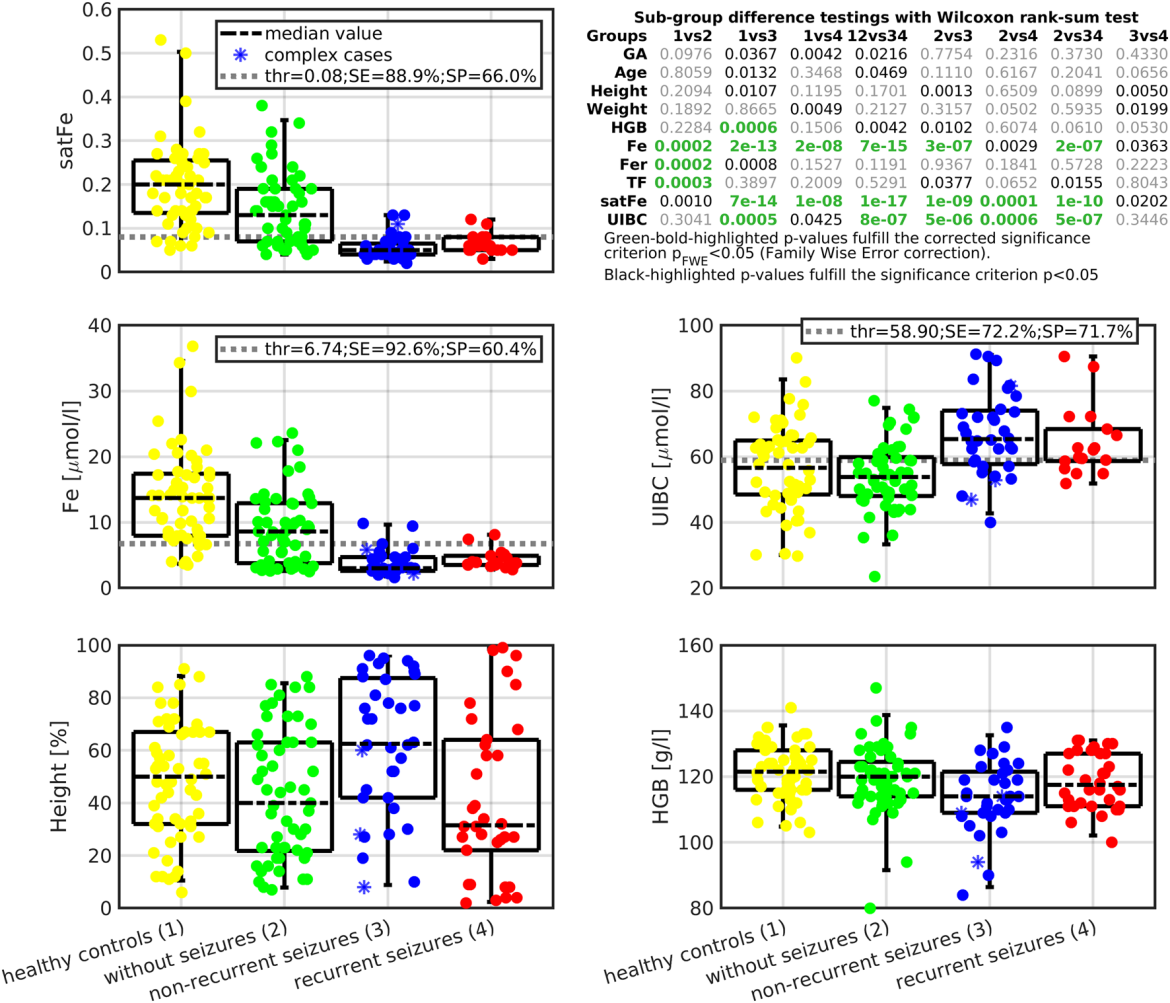
Between-group differences at the univariate level. The figure-embedded table summarizes between-group differences with highlighted significant findings. Graphs show value distributions for selected variables. Automatically enumerated discriminating thresholds (*dashed gray lines*) and corresponding SE and SP values are displayed for satFe, Fe, and UIBC variables, which demonstrated the strongest separation between groups. *1* healthy controls, *2* febrile patients without seizures, *3* febrile patients with non-recurrent FS, *4* febrile patients with recurrent FS, *GA* gestational age, *Age* age at the first febrile seizure attack, *Height* height percentile, *Weight* weight percentile, *HGB* hemoglobin, *Fe* serum iron concentration, *Fer* serum ferritin concentration, *FS* febrile seizures, *TF* serum transferrin concentration, *satFe* iron saturation, *UIBC* unsaturated iron-binding capacity, *thr* threshold, *SE* sensitivity, *SP* specificity.

Between-group differences were evaluated with the Wilcoxon rank-sum test for each examined variable (critical threshold value *p*_*FWE*_ < *0.05*; FEW—family-wise error correction; non-corrected p < 0.05 was considered as a trend in the data). For variables demonstrating significant differences between case and control groups, a maximal sum of SE + SP defined the variable-specific separating threshold (Fig. 1). The healthy control group was not included in the SE + SP estimations, as healthy children without fever do not seek medical attention. The SE + SP sum is proportional to a minimal Euclidean distance to the ideal *“gold standard”* classifier, i.e., the classifier with SE = 100% and SP = 100%, in the receiver operating characteristics (Fig. 4b).

Pearson cross-correlation analysis (*r*) investigated the presence of mutual linear relationships between variables (critical value *|r|*> *0.26 ≈ p* < *0.001* for 162 samples).

A univariate analysis does not usually reach the gold standard classifier property. As the blood and demographic screenings provide a low cross-correlated multi-dimensional dataset of “independent” variables, several data analysis approaches utilizing step-wise linear regression were designed to find a multivariate linear mixture model (Eq. (1)) that increases the SE + SP to FS risk or recurrence and gets closer to the gold standard classifier.

1 $${\textbf{y}}\, = \,{\textbf{x}}_{0} \, + \,\beta_{1} {\textbf{x}}_{1} \, + \, \cdots \, + \,\beta_{n} {\textbf{x}}_{n} \, + \,\boldsymbol{\varepsilon}$$

The vector ***x***_*0*_ is the constant member and the vector **ϵ** is Gaussian random noise. Vectors ***x***_*m*_ where index *m ∈ {1, 2,…,n}* represent *n* variables (i.e., variables derived from the blood screening or demographic variables) significantly contributing (*p* < 0.05) to the expected signal ***y***. Coefficients *β*_*m*_ define magnitudes of contributions. The crucial part of linear mixture modeling is the definition of the expected signal ***y***.

Three models (i.e., *model*_*1*_, *model*_*2,*_ or *model*_*3*_) with three different expected signals (i.e., ***y***_*1*_, ***y***_*2,*_ or ***y***_*3*_) were designed and tested. In *model*_*1*_, ***y***_*1*_ equals 0 at positions of healthy controls, equals 1 at positions of patients without FS, and 2 at positions of patients with FS. In the *model*_*2*_, only patients were considered, and ***y***_*2*_ equals 0 at positions of patients without FS and 1 at positions of patients with FS. In *model*_*3*_, only patients with FS were considered, and ***y***_*3*_ equals 1 at positions of patients with non-recurrent FS and equals 2 at positions of patients with RFS. Model-specific Wilcoxon rank-sum test, SE, SP, and the separating threshold maximizing the SE + SP sum were evaluated in the same fashion as for the univariate approach while getting closer to the gold standard classifier was the set goal.

Model_1_ and model_2_ represent two concurrent models potentially separating non-seizure and seizure patients with high SE and SP. Therefore, we tested whether an orthogonal projection (*f*) of both models into one bi-linear model ***y***_*12*_ (Eq. (2)) can even increase the SE and SP and improve the developing classifier. Two scalar separating thresholds y_1_ and y_2_ were again identified by maximizing the SE + SP sum.

2 $${\textbf{y}}_{12} \, = \,f\left( {{\textbf{y}}_{1} ,\,{\textbf{y}}_{2} } \right)$$

Continuous biological factors, such as age, gestational age, height percentile, and weight percentile, were additional inputs for the linear mixture modeling via the step-wise linear regression for model_1_, model_2,_ and model_3_. For model_3_, maximal body temperature and sodium and vitamin D concentrations were additional input variables in the regression analysis. Categorical biological factors should be spread uniformly over the dataset to guarantee a fair design of any classifier. Sex was distributed equally in the control groups. However, FS and RFS demonstrated higher prevalence and incidence in males. Therefore, we employed the adaptive synthetic sampling approach matching the number of female samples in the case (FS and RFS) groups to minimize the risk of imbalanced learning^13,14^. As initial conditions were randomized, each model training was repeated 5000 times to test and guarantee model stability and reliability. Moreover, sex was also used as a binary input variable in the regression.

The sample size of our dataset was limited. To test dataset power to establish stable FS risk and recurrence model/s, we have permutatively down-sampled the dataset to 90%, 80%, 70%, 60%, and 50% of its original size, while intra-group sex distributions remained unchanged. Again, the adaptive synthetic sampling matched the number of female samples in the case groups. Model training was 5000 times repeated for each dataset size. Objective measures assessing model/s’ stability and reliability were as follows: (i) frequency occurrence of the most common model (a priori defined by the full 100% dataset size); averages and variances of (ii) regression coefficient; (iii) explained variance; (iv) Pearson correlation coefficient between modeled and predicted signal ***y*** (Eq. (1)); (v) between-group separating threshold determined via the SE + SP sum maximization; and (vi) SE and SP. In under-sampled datasets, the SE and SP were assessed for selected (training) and unselected (testing) data points.

### Data and computer code availability and license statement

Raw input anonymized data and MATLAB language script (written in version R2018b) making statistical testing and deriving the regression models are available under the GNU General Public License version 3 at: https://github.com/umn-milab/febrile-seizure-blood-models (release r20231005).

Tools for cross-correlation analysis are available under the same license at: https://www.mathworks.com/matlabcentral/fileexchange/74204-corrplotg.

The MATLAB basic programming environment, MATLAB Statistics, Machine Learning Toolbox, and Econometrics Toolbox licenses need to be available to an end-user for full program compatibility.

The MATLAB implementation of the adaptive synthetic sampling is available in the ADASYN toolbox under the copyright^©^ 2015, Dominic Siedhoff: https://www.mathworks.com/matlabcentral/fileexchange/50541-adasyn-improves-class-balance-extension-of-smote.

## Results

Iron status results and demographics are summarized in Table 1. The prospective enrollment revealed a 2.5-fold higher incidence of FS and 3.25-fold of RFS in males than females, respectively. Control groups showed balanced sex distributions. Complex FS were all non-recurrent and occurred in three children (5.7%). Family history in the first-degree relatives for FS was positive in four cases (two females; 7.6%), who all presented with simple non-recurrent FS. Family history for epilepsy was positive in one male (1.9%) with simple RFS. Peak body temperature did not differ between FS subgroups. The EEG was recorded after the seizure and did not show a pathological finding in any case. In the follow-up, none of the study participants was treated for ID or anemia.

### Univariate between-group differences

Figure 1 shows significant between-group differences or trends for single variables. Group-specific demographics with iron status are in Table 1. Serum Fe, satFe, and UIBC were the only three variables demonstrating a significant difference between control and case groups (Fig. 1). The automatically enumerated thresholds with corresponding SE and SP are presented in Fig. 1. There were no significant differences for FS case subgroups at the single-variable level (Fig. 1). The significant difference in Fer levels was only between afebrile healthy controls and febrile children without seizures. The visualization of control and case groups for the single variables is shown in Fig. 1. Within-group differences were present in healthy controls when divided based on sex. The median and interquartile range (IQR, defined as 25–75% percentiles) of iron concentration was 10.4 (7.9–14.2) μmol/l in males and 15.3 (10.7–20.2) μmol/l in females (p = 0.021); iron saturation was 0.15 (0.12–0.24) in males; 0.21 (0.18–0.26) in females (p = 0.032). No other sex-related within-group differences were observed.

Serum electrolytes and vitamin D did not differ between FS and RFS groups. Sodium concentrations were 133 (130–137) mmol/L in FS and 133 (131–138) mmol/L in RFS. Vitamin D concentrations were 89.9 (46.9–135.1) nmol/L in FS and 77.0 (45.3–105.2) nmol/L in RFS.

### Linearly dependent variables

**Figure 2:**
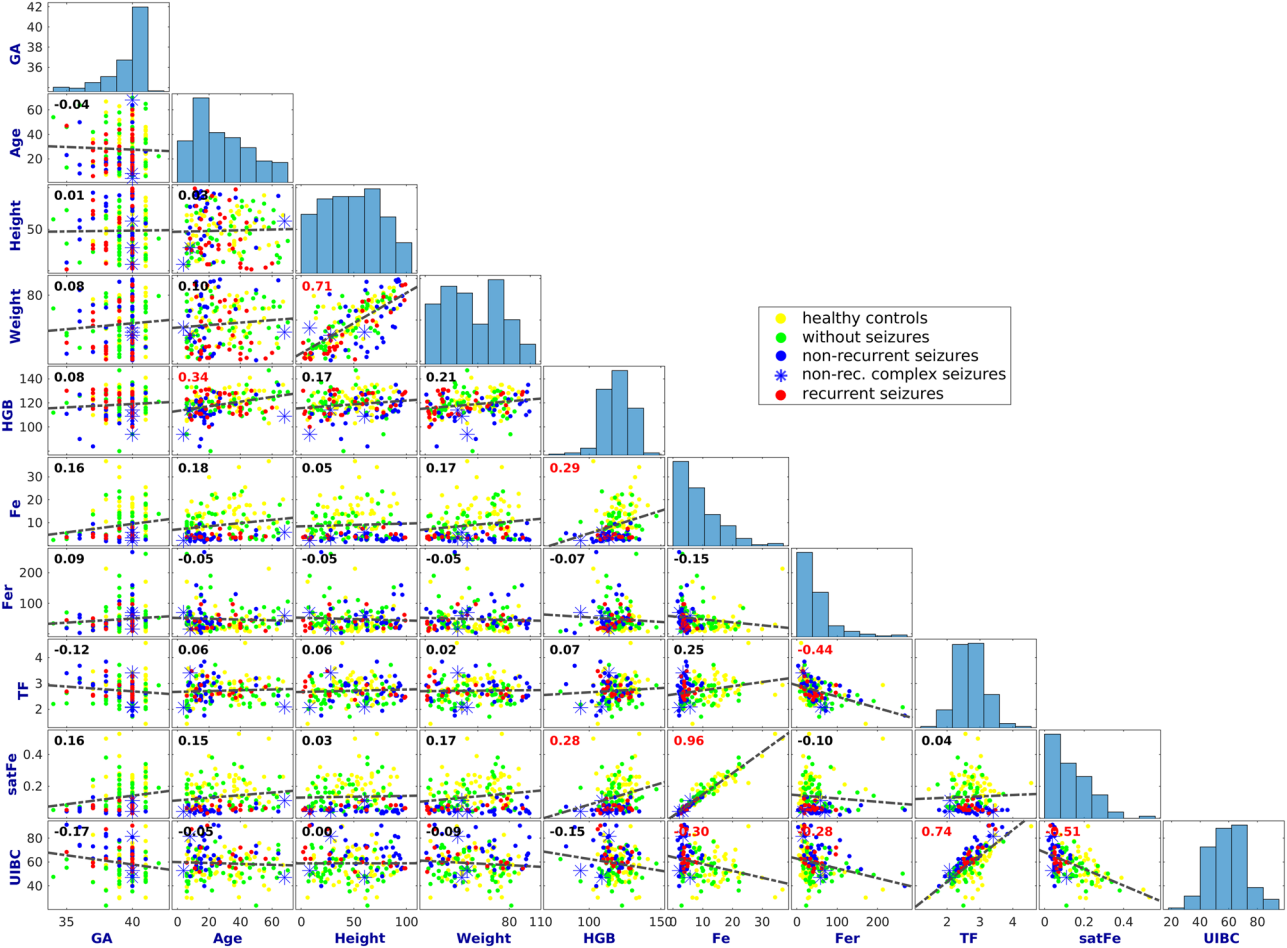
Cross-correlation matrix plot for investigated variables. Value in the upper-left corner of each plot is the Pearson correlation coefficient (r) for corresponding variable pairs. Value r is red-highlighted for the significant coefficient with p < 0.001. The correlation regression line is presented as a *black dashed line*. The values at x- and y-axes are fixed for each variable across the plot. Histograms at the main plot diagonal display the value distribution for each corresponding variable. *GA* gestational age (weeks), *Age* age at the first febrile seizure attack, *Height* height percentile, *Weight* weight percentile, *HGB* hemoglobin, *Fe* serum iron concentration, *Fer* serum ferritin concentration, *TF* serum transferrin concentration, *satFe* iron saturation, *UIBC* unsaturated iron-binding capacity.

As expected, height and weight percentiles were linearly dependent. In addition, several blood iron status variables were mutually cross-correlated. Demographics and iron status were not significantly correlated, except for the positive correlation between age and hemoglobin. A detailed view of the cross-correlation analysis is shown in Fig. 2. Simultaneously, we did not observe any clear non-linear relationships between variables (Fig. 2), which would suggest a potential necessity for the non-linear transformation of some variable/s before further linear mixture modeling.

### Multivariate linear models maximizing between-group differences

Multivariate linear mixture models with enhanced separating properties between case and control groups (i.e., model_1_ or model_2_) or between case sub-groups (i.e., model_3_) were defined.

**Figure 3:**
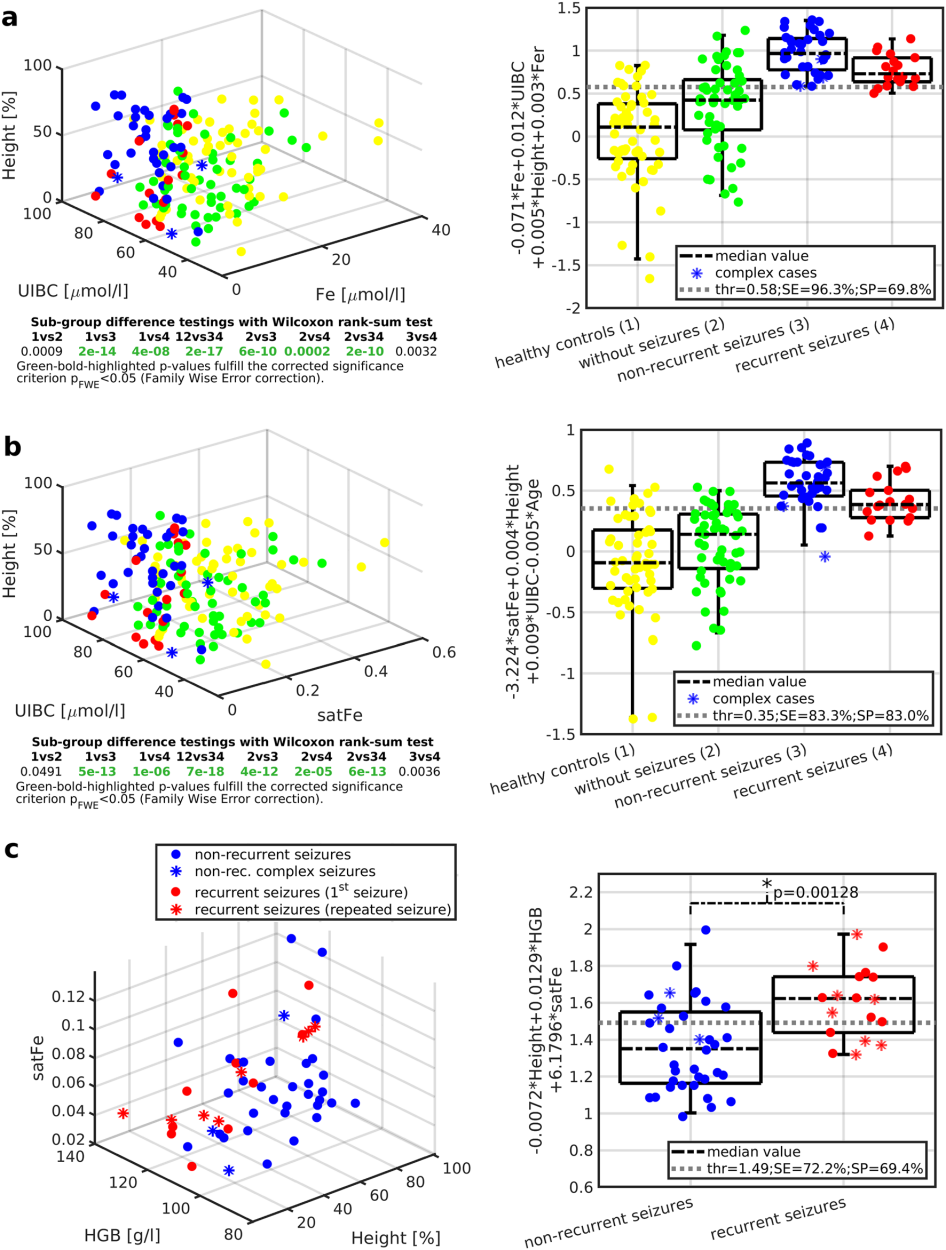
Between-group differences with multivariate linear mixture models. *Left-sided panels*: (**a-b**) represent dataset 3D visualizations in the space of three significant variables (in figure (**a**) height, UIBC, Fe; in figure (**b**) height, UIBC, and satFe) with p-values for respective between-group comparisons under each panel; **c** shows linear dependence between height percentile and HGB evaluated with Pearson correlation coefficient (r) for subgroups of patients with non-recurrent and recurrent febrile seizures. *Right-sided panels*: (**a-b**) show distributions of regressed values for all investigated groups, **c** for subgroups of patients with non-recurrent and recurrent febrile seizures. Automatically enumerated discriminating thresholds are shown with *dashed gray lines*; corresponding SE and SP values for separation properties of control and case groups are based on model_1_ (**a**), model_2_ (**b**), model_3_ **(c).** Model equations are displayed in the y-axis label descriptions. *1* healthy controls, *2* febrile patients without seizures, *3* febrile patients with non-recurrent FS, *4* febrile patients with recurrent FS, *Fe* serum iron concentration, *satFe* iron saturation, *Fer* serum ferritin concentration, *Age* age at the first febrile seizure attack, *Height* height percentile, *FS* febrile seizures, *UIBC* unsaturated iron-binding capacity, *HGB* hemoglobin, *thr* threshold, *SE* sensitivity, *SP* specificity, *p-values were evaluated with the Wilcoxon rank-sum test.

The model_1_ (Eq. (3), Fig. 3a, Table 2) identified the significant contribution of four linearly mixture variables (i.e., Fe, UIBC, height percentile, and Fer) forming a predicted signal **y**_p1_ with increased separating properties (SE = 95.49 ± 1.61%, SP = 69.43 ± 1.15%) between non-seizure and seizure patients with the separating threshold 0.5744 ± 0.0317. The quantitative characteristics of the estimated model_1_ (Eq. (3)) were as follows: F-value F = 38.41 ± 0.66, root mean square error RMSE = 0.6239 ± 0.0024, explained variance R^2^ = 46.04 ± 0.42%, and Pearson correlation coefficient r between the modeled signal **y**_1_ and predicted signal **y**_p1_ r = 0.643 ± 0.000. Means, including variances of derived regression coefficients, are listed in Table 2.

3 $$\mathbf{y}_{1} \propto {\mathbf{y}}_{p1} = \, - 0.071*{\mathbf{Fe}} + 0.012*{\mathbf{UIBC}} + 0.005*{\mathbf{Height}} + 0.003*{\mathbf{Fer}}$$

**Table 2:** Quantitative characteristics and stability of identified multivariate linear mixture models tested on full and undersampled dataset.

	Dataset size	100%	90%	80%	70%	60%	50%
Model1	Model detection rate [%]	*66.0	*20.8	11.2	6.4	3.2	2.2
	Total number of identified models	6	61	90	121	142	160
	Height regression coefficient	0.0049 ± 0.0002	0.0051 ± 0.0007	0.0052 ± 0.0008	0.0055 ± 0.0009	0.0059 ± 0.0011	0.0067 ± 0.0012
	Fe regression coefficient	− 0.0715 ± 0.0005	− 0.0702 ± 0.0025	− 0.0687 ± 0.0035	− 0.0684 ± 0.0049	− 0.0664 ± 0.0053	− 0.0654 ± 0.0074
	Fer regression coefficient	0.0025 ± 0.0001	0.0028 ± 0.0003	0.0031 ± 0.0004	0.0033 ± 0.0005	0.0037 ± 0.0007	0.0040 ± 0.0007
	UIBC regression coefficient	0.0119 ± 0.0004	0.0129 ± 0.0010	0.0136 ± 0.0015	0.0145 ± 0.0017	0.0153 ± 0.0023	0.0165 ± 0.0028
	F-statistics	38.41 ± 0.66	35.00 ± 2.43	31.54 ± 2.92	28.96 ± 3.31	25.45 ± 3.41	23.17 ± 3.83
	Root mean square error	0.6239 ± 0.0024	0.6221 ± 0.0094	0.6206 ± 0.0128	0.6162 ± 0.0163	0.6144 ± 0.0197	0.6012 ± 0.0247
	Explained variance *R*^*2*^ [%]	46.04 ± 0.42	46.61 ± 1.62	46.75 ± 2.20	47.95 ± 2.75	48.61 ± 3.28	50.92 ± 4.04
	Pearson correlation (***y***_***1***_ vs ***y***_***p1***_)	0.643 ± 0.000	0.646 ± 0.012	0.648 ± 0.017	0.656 ± 0.021	0.663 ± 0.026	0.677 ± 0.031
	Non-seizure/seizure separating threshold	0.5744 ± 0.0317	0.6853 ± 0.0793	0.7355 ± 0.1091	0.8442 ± 0.1372	0.9531 ± 0.1674	1.0655 ± 0.2197
	Training: sensitivity	95.49 ± 1.61	93.90 ± 4.98	95.51 ± 4.60	92.68 ± 5.92	91.39 ± 6.18	93.40 ± 6.53
	Training: specificity	69.43 ± 1.15	70.90 ± 4.15	68.95 ± 5.25	72.24 ± 6.39	73.91 ± 7.31	71.25 ± 8.07
	Testing: sensitivity		87.32 ± 15.00	89.65 ± 12.12	84.72 ± 12.09	80.26 ± 13.38	83.27 ± 12.29
	Testing: specificity		67.36 ± 18.25	65.29 ± 14.61	66.71 ± 10.79	67.45 ± 10.67	66.43 ± 9.29
Model2	Model detection rate [%]	*100.0	*72.0	*50.9	*24.9	14.2	6.8
	Total number of identified models	1	44	64	127	160	209
	Age regression coefficient	− 0.0050 ± 0.0002	− 0.0052 ± 0.0007	− 0.0056 ± 0.0009	− 0.0060 ± 0.0010	− 0.0064 ± 0.0011	− 0.0072 ± 0.0014
	Height regression coefficient	0.0036 ± 0.0002	0.0036 ± 0.0005	0.0038 ± 0.0006	0.0040 ± 0.0007	0.0043 ± 0.0008	0.0048 ± 0.0010
	satFe regression coefficient	− 3.2236 ± 0.0455	− 3.1911 ± 0.2123	− 3.1108 ± 0.2796	− 3.0829 ± 0.3491	− 2.9630 ± 0.3964	− 2.8432 ± 0.4344
	UIBC regression coefficient	0.0093 ± 0.0003	0.0094 ± 0.0011	0.0098 ± 0.0015	0.0100 ± 0.0016	0.0108 ± 0.0019	0.0113 ± 0.0020
	F− statistics	28.82 ± 0.63	25.12 ± 2.25	22.85 ± 2.59	21.00 ± 3.00	19.20 ± 3.24	17.28 ± 3.37
	Root mean square error	0.3620 ± 0.0019	0.3638 ± 0.0076	0.3642 ± 0.0097	0.3601 ± 0.0123	0.3558 ± 0.0150	0.3509 ± 0.0175
	Explained variance *R*^*2*^ [%]	47.38 ± 0.54	47.47 ± 2.10	48.01 ± 2.69	49.13 ± 3.41	51.02 ± 4.08	53.10 ± 4.60
	Pearson correlation (***y***_***2***_ vs ***y***_***p2***_)	0.660 ± 0.000	0.662 ± 0.015	0.667 ± 0.020	0.674 ± 0.026	0.691 ± 0.031	0.704 ± 0.034
	Non− seizure/seizure separating threshold	0.3495 ± 0.0261	0.3657 ± 0.0899	0.3779 ± 0.1170	0.4135 ± 0.1332	0.4652 ± 0.1607	0.4906 ± 0.1730
	Training: sensitivity	83.53 ± 1.04	81.15 ± 4.19	83.30 ± 4.35	80.72 ± 5.91	82.28 ± 6.42	85.87 ± 6.79
	Training: specificity	82.89 ± 0.92	86.06 ± 4.14	84.81 ± 4.94	88.90 ± 5.16	89.72 ± 5.66	88.30 ± 7.02
	Testing: sensitivity		75.60 ± 18.66	75.50 ± 13.62	71.20 ± 12.15	70.69 ± 10.81	72.76 ± 10.10
	Testing: specificity		81.14 ± 15.07	78.56 ± 12.53	81.53 ± 9.97	79.77 ± 10.16	77.35 ± 11.19
Model3	Model detection rate [%]	*51.5	28.4	10.4	4.6	2.0	1.1
	Total number of identified models	15	73	203	293	383	506
	Height regression coefficient	− 0.0072 ± 0.0005	− 0.0070 ± 0.0007	− 0.0079 ± 0.0011	− 0.0080 ± 0.0012	− 0.0083 ± 0.0012	− 0.0088 ± 0.0012
	HGB regression coefficient	0.0129 ± 0.0009	0.0136 ± 0.0013	0.0153 ± 0.0022	0.0158 ± 0.0024	0.0171 ± 0.0028	0.0179 ± 0.0028
	satFe regression coefficient	6.1796 ± 0.5323	6.1236 ± 0.8455	6.8889 ± 1.3212	7.0798 ± 1.4197	7.8790 ± 1.7529	9.1360 ± 2.7797
	F-statistics	8.24 ± 0.83	7.41 ± 1.30	8.79 ± 2.07	8.48 ± 2.31	8.60 ± 2.54	10.17 ± 3.67
	Root mean square error	0.4182 ± 0.0055	0.4130 ± 0.0095	0.4068 ± 0.0148	0.3917 ± 0.0178	0.3799 ± 0.0218	0.3615 ± 0.0300
	Explained variance *R*^*2*^ [%]	26.04 ± 1.93	26.37 ± 3.35	32.33 ± 4.90	35.13 ± 5.75	40.08 ± 6.62	47.23 ± 8.58
	Pearson correlation (***y***_***3***_ vs ***y***_***p3***_)	0.441 ± 0.005	0.457 ± 0.033	0.495 ± 0.046	0.533 ± 0.053	0.577 ± 0.057	0.630 ± 0.067
	Non-recurrent/recurrent seizure separating threshold	1.4001 ± 0.0999	1.4947 ± 0.1410	1.6849 ± 0.2406	1.7372 ± 0.2945	1.9210 ± 0.3366	2.0830 ± 0.3144
	Training: sensitivity	83.86 ± 7.67	86.15 ± 10.19	88.11 ± 11.57	91.45 ± 10.93	92.50 ± 8.92	92.03 ± 9.59
	Training: specificity	58.44 ± 6.69	60.50 ± 6.80	64.23 ± 9.26	66.54 ± 10.81	69.81 ± 10.25	76.00 ± 10.77
	Testing: sensitivity		73.33 ± 44.24	69.80 ± 30.16	74.70 ± 28.62	74.29 ± 26.08	69.81 ± 24.57
	Testing: specificity		45.18 ± 27.23	44.85 ± 19.98	42.13 ± 17.47	46.57 ± 14.52	49.61 ± 12.24

All values were averaged from utilized 5000 iterations with randomized initial conditions. Values are represented as mean ± standard deviation among the iterations. In a majority of the listed quantitative measurements, the mean values are quite stable and standard deviation increases as the dataset is more undersampled.

*The bold highlighted “Model detection rate” represents that the model with listed regression coefficients has been the most often identified as the best model characterizing the data among the iterations.

The adaptive synthetic sampling matched the number of female samples in the case groups to minimize the risk of the imbalanced learning within each modeling iteration.

The separating threshold has been identified by maximizing sum of sensitivity and specificity. Then, the classifying sensitivity and specificity have been tested on the training dataset itself and on the training dataset (i.e., the samples excluded from the training due to dataset undersampling).

Single-subject predicted y_p1_ values significantly separated all examined groups between themselves except for case sub-groups, and control subgroups (Fig. 3a).

The model_2_ (Eq. (4), Fig. 3b, Table 2) identified the significant contribution of four linearly mixture variables (i.e., satFe, UIBC, height percentile, and Age) forming a predicted signal **y**_p2_ with increased separating properties (SE = 83.53 ± 1.04%, SP = 82.89 ± 0.92%). The quantitative characteristics of the estimated model_2_ (Eq. 4) were as follows: F = 28.82 ± 0.63, RMSE = 0.3620 ± 0.0019, R^2^ = 47.38 ± 0.54%, and r = 0.660 ± 0.000. Means, including variances of derived regression coefficients, are listed in Table 2.

4 $$\mathbf{y}_{2} \propto {\mathbf{y}}_{p2} = \, - 3.224*{\mathbf{satFe}} + 0.004*{\mathbf{Height}} + 0.009*{\mathbf{UIBC}} - 0.005*{\mathbf{Age}}$$

Same as the model_1_, single-subject predicted y_p2_ values significantly separated all examined groups between themselves except for case sub-groups and control sub-groups (Fig. 3b).

**Figure 4:**
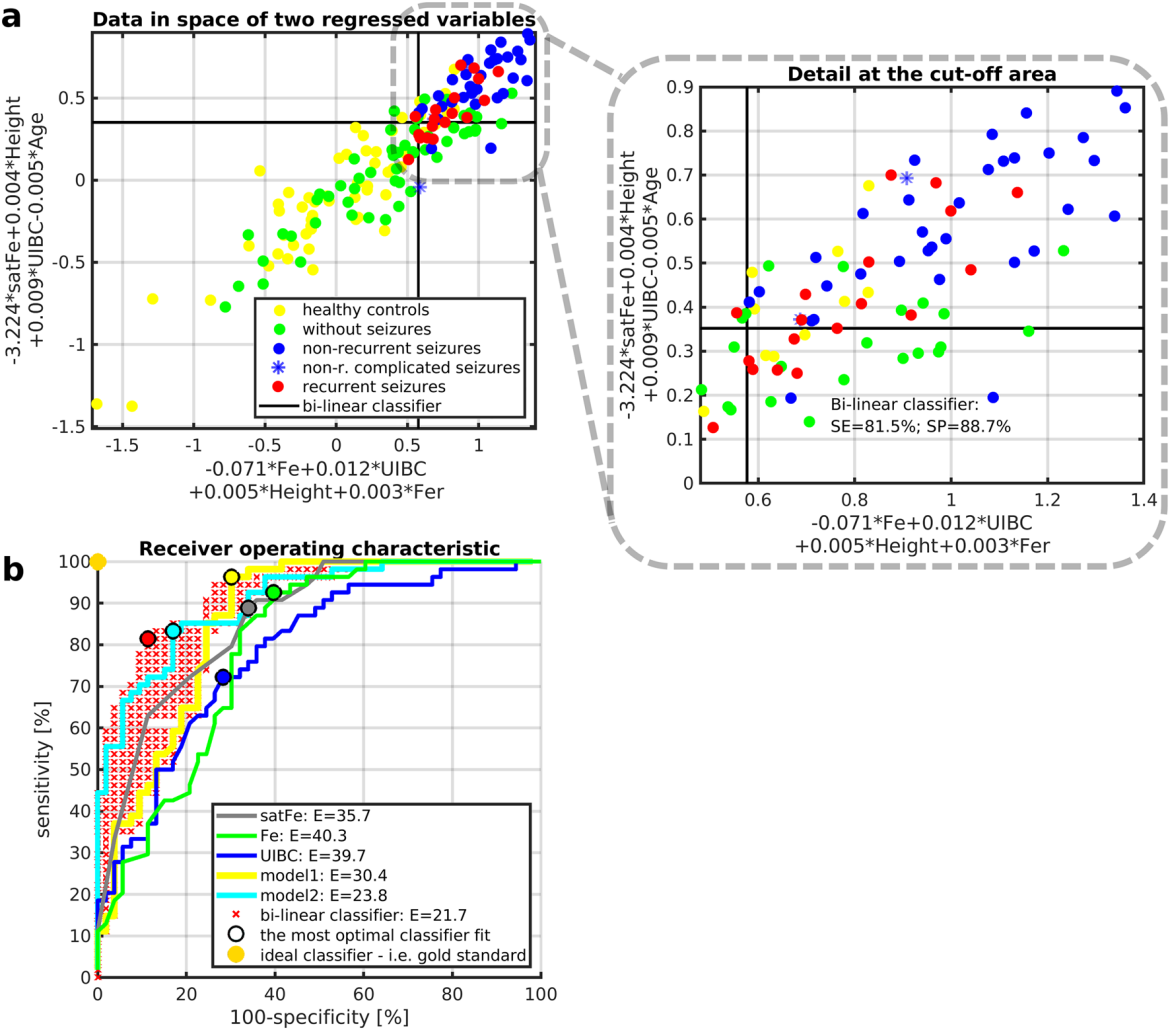
Increased specificity of the case group separation and receiver operating characteristics while combining model_1_ and model_2_. **(a**) Visualization of the mutual model_1_ (x-axis)—model_2_ (y-axis) projection for all investigated groups. Right panel shows the zoomed-in area (*delimited by dashed grey line*) of the upper-right quadrant. The bi-linear classifier represents the thresholds of each separate model_1_ and model_2_ derived from data distributions shown in Fig. 3a,b. Thresholds are visualized as black solid lines. (**b**) Receiver operating characteristics and Euclidean distance (E) between an ideal “gold standard” classifier and the optimal classifier fit for the corresponding model/variable. *Fe* serum iron concentration, *satFe* iron saturation, *Height* height percentile, *Age* age at the first febrile seizure attack, *UIBC* unsaturated iron-binding capacity, *HGB* hemoglobin, *thr* threshold, *SE* sensitivity, *SP* specificity, *ROC* receiver operating characteristics.

The mutual orthogonal projection (Eq. (2)) of model_1_ (Eq. (3)) and model_2_ (Eq. (4)) formed a bi-linear classifier providing the strongest separating properties (SE = 81.5%, SP = 88.7%; Fig. 4a).

All three presented linear mixture models (i.e., model_1_, model_2_, and bi-linear model_1_-model_2_ classifier; Fig. 3a,b, and 4a) improved separating properties and predictive power to FS risk when compared to the univariate analysis (Fig. 4b). The bi-linear classifier demonstrated the lowest Euclidean distance to the gold standard classifier (Fig. 4b).

Model_3_ (Eq. (5), Fig. 3c, Table 2) estimated a trivariate model (i.e., height percentile, HGB and satFe) forming a predicted signal **y**_p3_ with separation properties (p = 0.00128), which improved predictive power to FS recurrence when compared to model_1_ (p = 0.0032) or model_2_ (p = 0.0036; Fig. 3), or to univariate trends (height percentile p = 0.0050; weight percentile p = 0.0199; satFe p = 0.0202; and Fe p = 0.0363; Fig. 1). Quantitative characteristics of the model_3_ (Eq. 5) were as follows: F = 8.24 ± 0.83, RMSE = 0.4182 ± 0.0055, R^2^ = 26.04 ± 1.93%, and r = 0.441 ± 0.005. Due to suboptimal model characteristics, the subgroup-specific y_p3_ values remained overlapping, and separating SE/SP were limited to 83.86 ± 7.67% / 58.44 ± 6.69% (Fig. 3c). Means, including variances of derived regression coefficients, are listed in Table 2.

5 $$\mathbf{y}_{3} \propto {\mathbf{y}}_{p3} = \, - 0.0072*{\mathbf{Height}} + 0.0129*{\mathbf{HGB}} + 6.1796*{\mathbf{satFe}}$$

The parameter sensitivity analysis on under-sampled datasets showed the stability of the proposed regression coefficients in all three models. Still, their standard deviation increased as the dataset got more under-sampled (Table 2). Similar mean and standard deviation properties were applied for the models’ RMSE, R^2^, Pearson correlation, SE, and SP (Table 2). Models’ F value decreased, and the separating threshold increased as the dataset got more under-sampled (Table 2). When the dataset was divided into training and testing sub-datasets, the SE and SP were slightly lower on the testing dataset than obtained on the training dataset. However, both measurements remained proportional (Table 2). Model_2_ was the most stable and reproducible model as it remained the most often detected model even if the dataset was under-sampled to 70% of its original size (Table 2). Simultaneously, no other model was detected for the original 100% dataset size (Table 2). Model_1_ remained reproducible and the most often detected when the dataset was under-sampled to 90% of its original size (Table 2). Model_3_ was stable and reproducible only for the dataset of the original 100% dataset size (Table 2).

In summary, the under-sampled datasets led to models with either a sub-set of significant variables or a full set of significant variables and additional tested variables. However, such models were suboptimal compared to our models_1–3_. The significant contribution of presented variables can be expected in all three investigated models, but a certain validation of models_1_ and _3_ would benefit from a larger dataset (Table 2).

## Discussion

We confirmed the previous findings in febrile seizure research, such as blood iron status association with the risk of FS and higher incidence of FS in males than females with fever. More importantly, we designed novel multivariate linear mixture models for a potential accurate risk prediction of FS risk and recurrence based on blood iron status and demographic data. The models and, specifically, the derived bi-linear classifier demonstrated high SE + SP to discriminate between children who developed seizures and those who stayed seizure-free during the febrile episode. The accurate FS risk prediction among children with fever bears an unimagined potential in managing FS, such as FS prevention and avoiding the related stress and anxiety from seizure and hospitalization. Although our data were from a single center and the sample size is relatively limited, we propose the application of similar approach relying on multivariate models and classifiers to predict the risk of FS or RFS.

Multiple predictors have been identified^1,2,6–11^, pointing towards the multifactorial etiology of FS. One of the common FS predictors was the presence of ID^8,11^. Iron is an essential nutrient for brain maturation and overall body growth with unprecedented indispensability during “critical periods” of accelerated brain development spanning ages 6 to 24 months^15–17^. Within this time, the brain is prone to structural and functional alterations that may manifest immediately or arise later in life in the form of epilepsy^18,19^, neurodevelopmental problems such as memory problems, learning deficit, poor attention span, intellectual disability, behavioral disturbance^15,19,20^, or even as various psychiatric disorders^6,20,21^. Although the peak onset of FS is concurrent with this time period^8^, the impact of altered blood iron status on brain iron status, and consequently on brain structure and function, is unclear.

The previous literature on the blood iron status and FS mainly reported the association of ID and FS^1,8,11,22^, with some studies demonstrating non-existing or even opposite association^8–10^. Our findings showed a strong association between blood iron status and FS. Lower serum Fe levels and higher UIBC were in febrile children with seizures compared to those without seizures or afebrile healthy controls. The sensitivity of the serum iron measures to distinguish between the group with and without FS was high. Still, the specificity of these tests was relatively low, limiting their applicability in the clinical setting. Therefore, we generated multivariate mixture models for the group separation to increase the specificity. The models yielded the equations using specific variables such as ferritin and UIBC, iron concentration, and saturation. But also, body height and age were factors applied in the model to predict FS, despite the comparable and non-significantly different distribution across groups. Body height, age, and iron are interrelated with increased iron requirements in infancy and early years of life^23–25^. ID usually associates with faster growth whenever iron demands for growth exceed intake^26^. In the first two years of life, the risk of negative iron balance and organ prioritization may negatively affect brain development. The prioritization of iron distribution, which favors RBC (i.e., erythropoiesis) over the brain, heart, and skeletal muscles^15,16^, implies that ID may result in impaired neurodevelopment presenting with various degree of intellectual disability. Moreover, the elevated ferritin accompanying inflammation as an acute phase reactant is sequestered and, thus, not available for erythropoiesis and other organ systems. This defense mechanism, which aims to restrict serum iron from utilization by pathogens or tumors^27^, may lead or further contribute to ID, resulting in an increased risk of FS. Therefore, blood screening with an eventual iron-rich diet or iron supplementation may be warranted to prevent FS and neurodevelopmental sequelae.

We demonstrated that the bi-linear classifier consisting of two multivariate mixture models for the group separation provided high sensitivity and much improved specificity compared to univariate assessments or the models applied separately. Thus, carefully weighing the study limitations, we consider that the bi-linear classifier based on the presented models may represent a practical screening tool to determine the FS risk in febrile children. However, the robustness of the bi-linear classifier needs to be verified with a larger and more geographically and racially diverse cohort providing updated model coefficients or an extended variable list, which may result in the SE and SP at the proximity of the gold-standard classifier.

None of our models identified sex as a significant variable, although we observed higher FS incidence in males, which further confirms the findings of previous studies^1,2,6,11^. Significant sex effects were not observed in iron status and demographics in the febrile group without seizures and FS subgroups. In the healthy control group, lower iron concentration and saturation were noted in males compared to females. In analogy, the male sex represents a risk factor for ID or ID anemia in infants and young children^23,24,26,28^. Moreover, sex may determine seizure susceptibility and type, as demonstrated in the animal model^20,21^. The sex difference or male overrepresentation in FS human studies is well documented^1,2,6,11^. In the Japanese population, the male sex was identified as one of the major predictors of FS recurrence^2^. Our study showed more frequent RFS in males. Sex hormones control many molecular and cellular processes in brain differentiation, including the modification of the neural response to stress or brain injury. Thus, the increased FS susceptibility in males is likely influenced by multiple factors, including iron status alteration.

Regarding FS recurrence, the unique trivariate model consisting of HGB concentration, body height percentile, and Fe saturation was derived. The model’s reasonable separation (i.e., SE + SP) and model reproducibility were suboptimal, requiring further improvement and additional variables to define a model with optimal FS recurrence predictive power.

We only utilized linear mixture modeling between investigated variables. It is possible that the proposed analysis may benefit from a non-linear transformation of some variables before the regression analysis. However, we consider that strategy of a lower potential for a marginal improvement on the current dataset as we have not observed any non-linear relationships between variables. When the dataset is enlarged, the training of a non-linear classifier in the space of the orthogonal model_1_-model_2_ projection may lead to an improved models’ prediction.

### Study limitations

A small and geographically limited Caucasian sample size represented the primary study constraint. Thus, using the full dataset for model regression with the subsequent classifier evaluation may lead to classifier overfitting in all derived and tested models. Therefore, a re-test of fixed models will be necessary at a fully independent and larger dataset that will enumerate and validate true models’ SE and SP.

Body height or weight percentile tables normalized for the Czech population may differ across nations, and slightly varying regression coefficients may be derived (i.e., β coefficients in Eqs. (2), (3), or (4)). Future multi-center experiments with diverse pediatric populations may re-test or derive regression coefficient expectations with a specific variance and define more generalizable models’ normative values.

Imbalanced sex distribution in case groups may bias our findings. The employed adaptive synthetic sampling was performed in an effort to minimize such a dataset effect. Future research needs to collect vitamin D samples in all investigated cases to rightfully determine its role.

Similar to the previous FS studies, Refs.^1,11^ the serum Fer levels may be elevated in various inflammatory conditions as ferritin is an acute phase reactant and marker of acute and chronic inflammation. Reference^26^ Although the ferritin levels were not significantly different across febrile groups of children with or without seizures, the influence on overall iron status during inflammatory conditions, mainly restricted serum iron utilization^26^, is noteworthy and may contribute to the FS development.

## Conclusion

We confirmed the relationship between iron status and FS with a higher incidence in males. More importantly, we proposed a novel approach to evaluate the FS risk in infants and young children with fever. First, multivariate linear mixture models were derived based on blood iron status and demographic variables. The approach emphasized between-group separation properties when height percentile and age were included in the iron status observation. Next, a bi-linear classifier consisting of two multivariate mixture models provided the optimal SE + SP for FS risk. Finally, we have designed an innovative trivariate model sensitive to FS recurrence, utilizing height percentile, hemoglobin, and Fe saturation. We also hypothesize that a future extension of the novel FS recurrence model about the vitamin D variable can substantially improve its sensitivity and specificity. Future multi-center studies with a larger and more geographically and racially diverse cohort will re-test and validate the robustness of derived models to prove or disclaim them as classifiers with predictive power to FS risk or recurrence.
